# Alzheimer's Disease Diagnosis Based on Cortical and Subcortical Features

**DOI:** 10.1155/2019/2492719

**Published:** 2019-03-03

**Authors:** Yubraj Gupta, Kun Ho Lee, Kyu Yeong Choi, Jang Jae Lee, Byeong Chae Kim, Goo-Rak Kwon

**Affiliations:** ^1^School of Information Communication Engineering, Chosun University, 309 Pilmun-Daero, Dong-Gu, Gwangju 61452, Republic of Korea; ^2^National Research Center for Dementia, Chosun University, 309 Pilmun-Daero, Dong-Gu, Gwangju 61452, Republic of Korea; ^3^Department of Biomedical Science, College of Natural Sciences, Chosun University, 309 Pilmun-Daero, Dong-Gu, Gwangju 61452, Republic of Korea; ^4^Department of Neurology, Chonnam National University Medical School, Gwangju 61469, Republic of Korea

## Abstract

Alzheimer's disease (AD) is a common neurodegenerative disease with an often seen prodromal mild cognitive impairment (MCI) phase, where memory loss is the main complaint progressively worsening with behavior issues and poor self-care. However, not all patients clinically diagnosed with MCI progress to the AD. Currently, several high-dimensional classification techniques have been developed to automatically distinguish among AD, MCI, and healthy control (HC) patients based on T1-weighted MRI. However, these method features are based on wavelets, contourlets, gray-level co-occurrence matrix, etc., rather than using clinical features which helps doctors to understand the pathological mechanism of the AD. In this study, a new approach is proposed using cortical thickness and subcortical volume for distinguishing binary and tertiary classification of the National Research Center for Dementia dataset (NRCD), which consists of 326 subjects. Five classification experiments are performed: binary classification, i.e., AD vs HC, HC vs mAD (MCI due to the AD), and mAD vs aAD (asymptomatic AD), and tertiary classification, i.e., AD vs HC vs mAD and AD vs HC vs aAD using cortical and subcortical features. Datasets were divided in a 70/30 ratio, and later, 70% were used for training and the remaining 30% were used to get an unbiased estimation performance of the suggested methods. For dimensionality reduction purpose, principal component analysis (PCA) was used. After that, the output of PCA was passed to various types of classifiers, namely, softmax, support vector machine (SVM), *k*-nearest neighbors, and naïve Bayes (NB) to check the performance of the model. Experiments on the NRCD dataset demonstrated that the softmax classifier is best suited for the AD vs HC classification with an F1 score of 99.06, whereas for other groups, the SVM classifier is best suited for the HC vs mAD, mAD vs aAD, and AD vs HC vs mAD classifications with the F1 scores being 99.51, 97.5, and 99.99, respectively. In addition, for the AD vs HC vs aAD classification, NB performed well with an F1 score of 95.88. In addition, to check our proposed model efficiency, we have also used the OASIS dataset for comparing with 9 state-of-the-art methods.

## 1. Introduction

Alzheimer's disease (AD) is the most usual neurodegenerative dementia and a rapidly growing health problem, which is the main reason for causing dementia in the elderly population [1]. A conclusive analysis can only be made postmortem and requires histopathological confirmation of neurofibrillary tangles and amyloid plaques. Early and correct diagnosis of AD is not only difficult but also vital from the perspective of future treatments.

Although there is currently no treatment for AD, there are several promising pharmacologic compounds in advanced stages of development, and it is expected that a treatment will be soon available. One of the factors that have been speculated to be the cause of lack of success in developing disease-modifying treatments thus far is the inability to correctly differentiate patients at the mild cognitive impairment (MCI) stage who will progress on to develop AD from those that have MCI symptoms that are due to other causes. A potentially promising treatment for AD may not be beneficial when administered to those whose MCI symptoms are not due to AD, causing an apparently promising treatment to be considered a failure. Another reason for distinguishing those patients with prodromal AD at the MCI stage is that interventions early in the course of the disease may help delay the onset and/or mitigate the risk of full-blown AD [2], whereas interventions later in the course of the disease may limit the disease but would not be able to reverse the pathology-induced neuronal loss after it has already occurred. Hence, diagnosis of presymptomatic AD in the MCI stage is a highly important task, which will become even more crucial and urgent when curative treatment becomes available. Such diagnosis can also be valuable for those whose MCI is due to causes other than AD, as these causes may be easier to determine and manage. Hence, neuroimaging improves the positive predictive value of the diagnosis and involves measurements by structural MRI to test medial temporal lobe atrophy (MTL) and positron emission tomography using fluorodeoxyglucose (FDG) or amyloid markers. MTL structures are fundamental for the creation of new memories and are centrally related to the growth of AD [3]. Specifically, MTA atrophy and allied episodic memory impairment are trademark features of AD, and they both progressively lessen over the course of the disease [4]. Normally, MTA is determined by using vertex-based [5], voxel-based [6], and ROI-based [7] approaches.

In this study, the focus is on binary and tertiary classification between AD, HC, mAD (MCI due to AD), and aAD (asymptomatic AD) using structural MRI. By using the atrophy measure from sMRI scans, the intensity and stage of the neurodegeneration can be determined. These studies include morphometric approaches, such as the region of interest (ROI) and volume of interest (VOI) of gray matter voxels for the automatic segmentation of sMRI images, and the sMRI volume measurement of the hippocampus and the medial progressive lobe [8]. Various machine-learning methods have been used to differentiate binary and tertiary classification between AD, HC, mAD, and aAD. As suggested in [9, 10], only using amyloid imaging biomarker may be less sensitive in tracing AD progression, mainly in the symptomatic stage, recent consensus statements have underlined the importance of a biomarker of neurodegeneration, which is a critical component of AD pathophysiology in the prodromal and early dementia stages, and also from [11], atrophy on sMRI reflects on cumulative loss and shrinkage of the neuropil [12–14]. These indicate that a volumetric measure of cortical thickness and subcortical volume is one essential biomarker for early detection of the AD. Cortical thickness analysis establishes as another widely accepted approach to measure gray matter atrophy in the AD, and cortical thinning has been found in MCI and AD. Despite evidence for subcortical amyloid and neurofibrillary tangle formation in AD [15], MRI research has drawn its attention to AD-related subcortical structure changes only recently. Advanced segmentation techniques now permit the quantification of subcortical volumes and provide the basis for subcortical shape analysis. Measurement of structural changes based on brain MRI scans has previously been used to classify AD patients versus cognitively normal (CN) subjects and to predict the risk of progression from MCI to the AD. Jha et al. [16] proposed a method for diagnosis of the AD using complex dual-tree wavelet principal coefficients transform (DTCWT). They have used two datasets, OASIS and ADNI, for the validation of their results. An extreme learning machine was used as a classifier, and an accuracy of 90.26%, sensitivity of 90.27%, and specificity of 90.27% were achieved for the 2D ADNI dataset, and an accuracy of 95.75%, a sensitivity of 96.59%, and a specificity of 93.03% were achieved for the OASIS dataset. Chyzhyk et al. [17] uses a lattice independent component analysis (LICA) technique for the feature section stage with combine kernel transformation of the data. Their approach improved the generalization of the dendritic computing classifiers. Then, they apply their proposed model on the OASIS dataset for classification of AD patients with normal subjects, and their model achieve 74.75% accuracy, 96% sensitivity, and 52.5% specificity. Khajehnejad et al. [18] uses the manifold-based semisupervised learning method for early diagnosis of the AD. They have used the OASIS dataset for experiment, and their proposed method yield accuracy of 93.86%, sensitivity of 94.65%, and specificity of 93.22%. Jha and Kwon [19] proposed an AD detection method using sparse autoencoder, scale conjugate gradient, and softmax output layer with fine-tuning. They have used the OASIS dataset and achieved an accuracy of 91.6%, 98.09% of sensitivity, and 84.09% of specificity. Islam and Zhan [20] proposed an ensemble of a deep convolutional neural network for early detection of AD. Here, they have used DenseNet-121, DenseNet-161, and DenseNet-169 deep learning models for classification of the OASIS dataset, and later, they have used their proposed ensemble model, which was fused with deep learning for classification of the same dataset. Their proposed method yields 93.18% of accuracy, 94% of precision, 93% of sensitivity, and 92% of F1 score. Farhan et al. [21] proposed a model, which uses two extracted features of the brain: first features are the volume of GM, WM, and CSF and second features are the (l h/rh) area of the hippocampus. In addition, later, they have used four different classifiers (SVM, MLP, j48, and ensemble of classifiers) to evaluate the classification accuracy. An ensemble of classifiers has got the high accuracy compared to other classifiers with 93.75% accuracy for combined features. Lama et al. [22] proposed a binary and a tertiary classification between AD, MCI, and NC using sMRI data from the ADNI dataset. A regularized extreme learning machine (RELM) was used as a classifier, and an accuracy of 77.30% for binary classification (AD vs NC) and 76.61% for tertiary classification (AD vs NC vs MCI) were obtained; cortical thickness features were used in the experiment. Based on a large subgroup of ADNI data, Cuingnet et al. [23] compared ten sMRI-based feature extraction techniques and their ability to distinguish between clinically relevant subject groups. These methods comprised five voxel-based techniques, three methods based on cortical thickness and two techniques based on the hippocampus. Optimum sensitivity and specificity values were (81%, 95%) for AD vs HC, (70%, 61%) for S-MCI vs P-MCI, and (73%, 85%) for HC vs P-MCI. Zhang et al. [24] proposed a multimodal classification approach by employing a multiple-kernel support vector machine (SVM) based on biomarkers including sMRI, PET, and cerebrospinal fluid (CSF) to distinguish AD (or MCI) and normal control (NC) subjects. For the binary classification (AD vs NC and MCI vs NC) results, their suggested model had high accuracy for AD classification, whereas for MCI classification, satisfactory accuracy was obtained. More recently, Cho et al. [25] conducted an experiment on 72 MCIc and 131 MCInc subjects using the incremental learning technique based on spatial frequency, which shows the representation of cortical thickness data. In addition, their proposed method yielded better results than the ten benchmark techniques for MCIc vs. MCInc classification as reported in [23], and a sensitivity of 63% and a specificity of 76% were obtained. Wolz et al. [26] used four different automated feature extraction techniques (namely, hippocampal volume, TBM, cortical thickness, and manifold-based learning) to analyze structural MRI data of 834 ADNI AD, MCI, and healthy control (CTL) subjects. The extracted features were used to compare the performance of two classifiers, namely, LDA and SVM, for AD classification and MCI prediction. The best accuracy for AD versus CTL classification was obtained by combining all extracted features and utilizing a LDA classifier, that is, an accuracy of 89% (sensitivity and specificity of 93% and 85%, respectively). Similarly, using combined features and the LDA classifier resulted in the highest accuracy of 68% (sensitivity and specificity of 67% and 69%, respectively) for classification of MCI-converter and MCI-stable subjects. When different feature types were studied individually, the TBM features yielded the best result.

Compared to earlier work, this study aims at establishing the enhancement in accuracy and constancy that can be attained by combining more than one MR-based feature. Here, we are going to investigate a combined feature of cortical thickness and subcortical volume in the AD and aAD with stable cognitive abilities compared to HC as well as in mAD which converted to AD. NRCD data were used to test the potential combination of diverse MR-based features for improving classification accuracy. For evaluation, all 326 NRCD dataset images provided by the dementia center of Chosun University hospital were used and NeuroI [27] which was developed at NRCD was also used. Specifically, the binary classifications AD vs HC, HC vs mAD, and mAD vs aAD and the tertiary classifications AD vs HC vs mAD and AD vs HC vs aAD were used. To this end, four representative classifiers are presented and compared, using an efficient feature selection approach, including SVM, *k*-nearest neighbors (KNN), naïve Bayes (NB), and softmax classifiers, for the multiclass classification of various stages of the AD progression.

## 2. Materials and Methods

### 2.1. Subjects

The data utilized in this research were obtained from the National Research Center for Dementia (NRCD). All the patients for whom preprocessed images were available were selected. The dataset contained 326 subjects: 81 AD subjects (39 males, 42 females; age ± SD = 71.86 ± 7.09 years, range = 56–83 years; education level = 7.34 ± 4.88, range = 0–18), 171 cognitively normal health control (HC) subjects (83 males, 88 females; age ± SD = 71.66 ± 5.43, range = 60–85; education level = 9.16 ± 5.54, range = 0–22), 39 patients with mAD (MCI who had converted to AD) (25 males, 14 females; age ± SD = 73.23 ± 7.09, range = 49–87; education level = 8.20 ± 5.19, range = 0–18), 35 patients with aAD (MCI who had not converted) (15 males, 20 females; age ± SD = 72.74 ± 4.82, range = 61–83; education level = 7.88 ± 6.30, range = 0–18).

Table 1 shows the demographics of the 326 study subjects. Statistical significant differences in demographics and clinical variables between the study groups were measured using Student's unpaired *t*-test. In this study, the significance level was set to 0.05, which is a standard alpha value. There were more females in all groups except for the mAD group. The education level was highly dissimilar in the pairwise comparisons between all other study groups. Compared to the patients in all other groups, AD patients had significantly a lower education level.

To obtain unbiased estimations of the performance, the set of contestants was randomly split into two parts in a 70/30 ratio for training and testing. The algorithm was trained on a training code, and the measures of the group performance were evaluated in terms of accuracy, sensitivity, specificity, precision, and F1 score using an independent test set.

### 2.2. MRI Acquisition

Standard 3T T1-weighted images were obtained using the volumetric 3D MPRAGE protocol with resolution 1 mm × 1 mm × 1 mm (voxel size). These were N4 bias correction images.

### 2.3. Feature Selection

**Figure 1:**
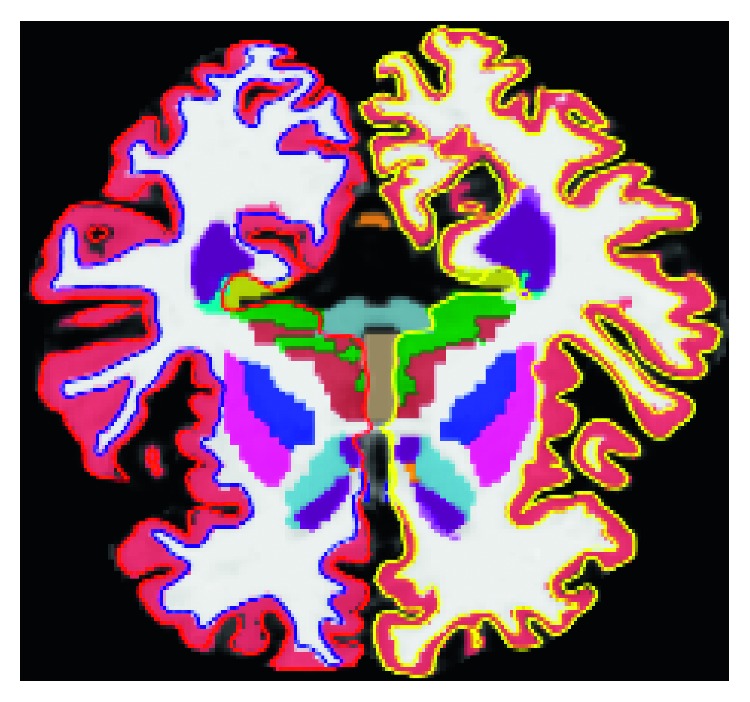
Cortical thickness showing left and right hemisphere of pial and white surface.

**Figure 2:**
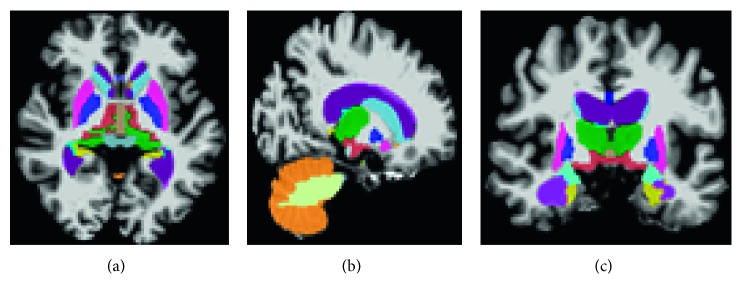
Subcortical brain segmentation.

Subcortical volumetric and cortical thickness measures have been widely used for classification purposes. From [28], we can say that cortical thickness is a direct index of atrophy and is, therefore, a potentially powerful candidate in the diagnosis of AD. In this experiment, cortical thickness and subcortical volume are extracted using Freesurfer (v5.3). In total, 110 features were extracted from 3D sMRI T1-weighted image. Freesurfer is a set of tools for analysis and visualization of cortical (Figure 1) and subcortical (Figure 2) brain imagining data. Freesurfer was designed around an automated workflow that includes several standard image processing phases which are necessary to achieve a final brain parcellation image within the subject's space; however, manual image editing is also allowed after each stage to ensure quality control (http://surfer.nmr.mgh.harvard.edu/fswiki). At first, a good quality of T1-weighted sMRI image volume is passed to the Freesurfer pipeline, and after that, Freesurfer performs a series of steps, start from motion artifact correction, affine transformation (12 degrees of freedom) to Talairach image space, nonuniform intensity normalization for intensity inhomogeneity correction, and removal of nonbrain tissues. The remaining brain image volume is intensity normalized to tie with the Freesurfer atlas image intensity histogram, which is followed by a nonlinear warping of the atlas brain image to subject brain image. A warped Atlas 3D brain image in the subject space is utilized in atlas-based tissue segmentation, for labelling subcortical brain structures, like brain stem, cerebellum, and cerebral cortex. The next step in Freesurfer is to generate topologically correct cortical surface representation per hemisphere, like gray-white matter segmentation [29], and the third segments of 34 ROIs based on anatomic landmarks [30].

**Figure 3:**
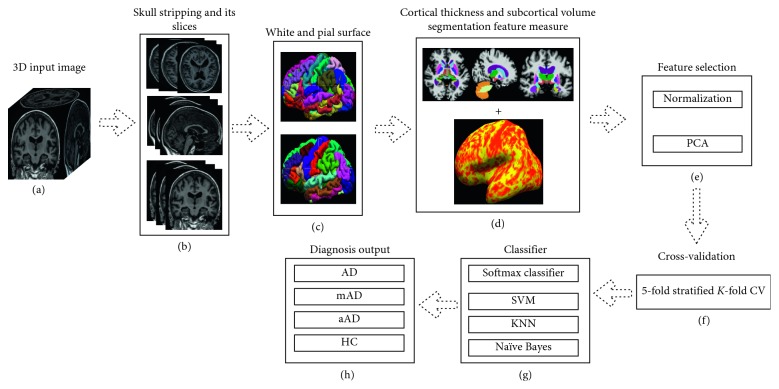
Workflow of proposed method: (a) 3D input image, (b) skull stripping, (c) white and pial surface, (d) cortical thickness and subcortical volume segmentation features, (e) feature selection (f) cross validation, (g) various classifiers, and (h) diagnosis output.

Freesurfer also provides the ability to construct a surface-based morphometry (SBM) for representations of the cortex, from which neuroanatomic volume, cortical thickness, and surface area can be derived. Cortical surface lies either at the WM/GM tissue interface or at the GM/CSF tissue interface. Each hemisphere's cortical surface representation is mapped automatically to a standard spherical coordinate system. Key components of the surface mapping include surface inflation with minimal metric distortion, topology correction, projection to spherical coordinates, and SBM warping to correctly align anatomical homologous points. Mapping to the standard spherical coordinate system defined by Freesurfer atlas brain allows for automated anatomical parcellation of cortex into gyral regions. Surface parcellation is then extended to GM volume, yielding parcellation of GM tissue sheet and regional cortical volumes. The precise matching of a morphologically homologous cortical location for each patient was obtained by plotting the atlas-based cortical surface on a sphere aligning the cortical patterns.  Table 2 presents an overview of the features calculated for all 326 available NRCD images. After the preprocessing stage, all the data extracted by Freesurfer were normalized to zero mean and unit variance for each feature, as shown in Figure 3 using standard scalar function of Scikit-learn library. That is, given the data matrix *X*, where rows represent subjects and columns represent features, the normalized matrix with elements *x*(*i*, *j*) is given by

(1) $$\begin{array}{c} X_{\text{norm},\left(i,j\right)}=\frac{x_{\left(i,j\right)}-\text{mean}\left(x_{j}\right)}{\text{std}\left(x_{j}\right)}, \end{array}$$

where *X*_*j*_ is the *j*^th^ column of the matrix (*X*). Subsequently, principal component analysis (PCA) was performed [31], which is a dimensionality reduction technique whereby the features are mapped onto a lower dimensional space. PCA is a feature selection process generating new features that are linear combinations of the initial features. PCA maps the data in a *d*-dimensional space to a new *k*-dimensional subspace with *k* *<* *d*. The new *k* variables are called principal components (PC), and each principal component has maximum variance eliminating the variance that is already accounted for in all succeeding components. Consequently, the first component has a larger variance than the components that follow. The principal components can be defined by the following equation:

(2) $$\begin{array}{c} {\text{PC}}_{i}=a_{1}b_{1}+a_{2}b_{2}+\cdots +a_{d}b_{d}, \end{array}$$

where PC_*i*_ is a principal component in *i*, *x*_*d*_ is an original feature in *d*, and *a*_*d*_ is a numerical coefficient of *x*_*d*_. The number of principal components is always less than or equal to the number of original observations.

**Figure 4:**
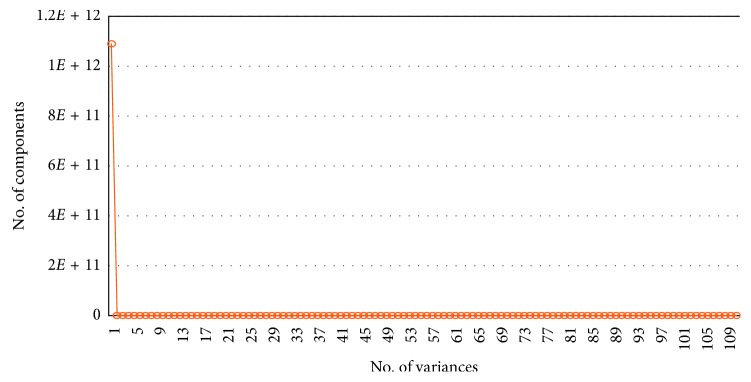
Number of principal components vs number of variances.

The obtained principal components for AD vs HC are shown in Figure 4. The number of components was determined by maintaining the variance greater than 99%. In total, there were 110 features; however, not all features were required for convergence. As can be seen in Figure 4, the first principal component achieved 99% of the variance compared to the other features. Therefore, the values that were obtained first were taken as a principal component for AD vs HC. Likewise, for other binary and tertiary classification, the same procedure was followed.

### 2.4. Classification Method

Once the sMRI-based features were extracted from the automated toolbox in both cortical and subcortical regions, feature vectors containing mean-centered voxel intensities were created combining all features. The classification approach we design is aimed to combine two sources of features: cortical thickness and subcortical volume. These features are used for global decision framework to discriminate AD with other groups. The overall diagram of the approach is presented in Figure 3. Here, our aim is early classification of an AD from other groups by using combined features of cortical thickness and subcortical volume of the same subjects. Our aim is to classify binary and tertiary groups. Moreover, classification is the problem of categorizing to which a new set of observation belongs, based on a training dataset whose group affiliation is already known. Moreover, we know that different classifiers follow a different mathematical function for classification. That is why we have used four best machine-learning classifiers, which are in practice like SVM and its variants, KNN, NB, and softmax classifiers, to know which classifier learns our model accurately to classify the different groups, and we also compared their results. All classification methods give a decision output.

### 2.5. SVM

**Figure 5:**
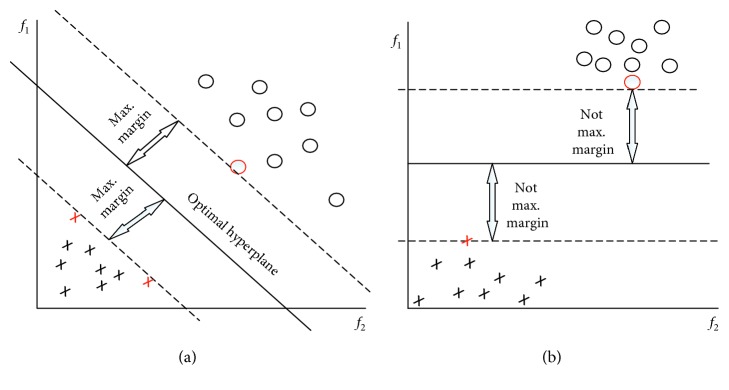
SVM optimal hyperplane.

This is the most common classification method to analyze sMRI data [23]. SVM [32] is the most commonly used algorithm in AD research for multivariate classification [26]. This method is based on choosing a critical point for the classification task. Support vectors are the elements of the data that are relevant in the splitting into two classes. The SVM algorithm determines the parameters of the decision function that maximizes the margin between the training examples and the class boundary, as shown in Figure 5. A number of nonlinear SVM approaches have also been used, such as kernel SVM and multikernel SVM [33].

The main concept of kernel techniques is to map the input data, which are linearly nonseparable into a higher dimensional feature space, where they are more likely to be linearly separable.

### 2.6. KNN

Cover and Hart proposed KNN in 1968 [34], which is one of the simplest machine-learning algorithms. It is an extension of the simple nearest neighbor technique. KNN classifies an unknown sample depending on the “vote” of the *k*-nearest neighbors rather than the single nearest neighbor.

The main steps of KNN implementation are as follows:

(1) Similarity assessment: the similarity between the test sample and each sample of the training set is calculated. In general, the resemblance can be measured by, for instance, the Euclidean distance, the Manhattan distance, the Jacquard similarity coefficient, and the correlation coefficient. Among these, the Euclidean distance method is the most widely used. For a given feature sample test(*x*_*j*1_, *x*_*j*2_,…, *x*_*ji*_) and training set feature train(*x*_*j*1_, *x*_*j*2_,…, *x*_*jn*_), the Euclidean distance is calculated as follows:
  (3) $$\begin{array}{c} d_{j}=\sqrt{\overset{n}{\underset{i=1}{\sum }}\left({\text{test}}_{ji}-\text{t}{\text{rain}}_{jn}\right)}, \end{array}$$
  where *n* is the number of feature vectors, *j* is the number of training and testing trials, and *d*_*j*_ is the Euclidean distance between the *j*^th^ sample of the training set and a test sample.
(2) In the second step, the neighbor's nearest distances should be determined and sorted in ascending order. There are several methods (like elbow method and thumb rule) which help us to achieve the best *k* value for KNN. The optimum *k* value will always vary depending on the dataset, so it should be as big as that its noises will not disturb the prediction highly and as low as that one factor will not dominate another. Then, the selection of the value will directly affect the classification result and should thus be carefully made.
(3) In the third step, the “vote and classify” method is applied, whereby the test sample is classified to a class according to the voting result of each category.

### 2.7. NB

NB is a machine-learning technique that has been utilized for over 50 years in biomedical informatics [35, 36]. These classifiers are a family of simple “probabilistic classifiers” based on Bayes' theorem with strong independence assumptions between their features. An NB classification model is easy to construct, with no complex iterative parameter approximation, which makes it particularly useful for large datasets. Despite its simplicity, the NB model often performs unexpectedly well and is widely used for classification because it often outperforms several more sophisticated classification approaches. Bayes' theorem is used to determine the posterior probability *p*(*c*/*x*) from *p*(*c*), *p*(*x*), and *p*(*x*/*c*). The NB classifier assumes that the effect of the parameter of a predictor (*x*) on a specified class (*c*) is autonomous compared with the other predictor's parameters. This assumption is called class-conditional independence:

(4) $$\begin{array}{c} p\left(\frac{c}{x}\right)=\frac{p\left(x/c\right)p\left(c\right)}{p\left(x\right)}, \end{array}$$

where *p*(*c*/*x*)=*p*(*x*_1_/*c*) *∗* *p*(*x*_2_/*c*) *∗*  ⋯  *∗* *p*(*x*_*n*_/*c*) *∗* *p*(*c*)  ·  *p*(*c*/*x*) is the posterior likelihood of the class (target), given the predictor (attribute); *p*(*c*) is the prior probability of the class; *p*(*x*/*c*) is the probability of the predictor given the class; and *p*(*x*) is the prior probability of the predictor.

### 2.8. Softmax Classifier

Softmax regression is a generalized form of logistic regression that can be used in multiclass classification problems where the classes are mutually exclusive. The earlier linear regression model [19, 37] produces continuous or unbounded *y* values. To proceed from arbitrary values *y*  *∈ℝ*^*c*^ to normalized probability values, *p*  ∈*ℝ*^*c*^ is estimated for a single instance and exponentiation and normalization are used as follows:

(5) $$\begin{array}{c} p_{i}=\frac{\exp\;y_{i}}{\sum_{c=1}^{c}\exp\;y_{c}}, \end{array}$$

where *i*, *c*  *∈*{1,…, *C*} is the range over the classes, *p*_*i*_ refers to the probabilities, and *y*_*i*_  and  *y*_*c*_ refer to the value of a single instance. This is called the softmax function, which takes the vector of arbitrary real-valued scores in *y* and transforms it into a vector of values ranging between 0 and 1 that later sum to 1. A model that converts the un-normalized values at the end of a linear regression to normalized probabilities for classification is called softmax classifier. Here, the softmax layer takes the learned representation *p*_*i*_ and interprets it to the output class. A probability score *p*_*i*_ is also assigned to the output class. The softmax classifier understands the scores inside the output vector *y* as the un-normalized log likelihoods for each specific class and then replaces the hinge losses with a cross-entropy loss that has the form:

(6) $$\begin{array}{c} L_{i}=-\log\left(p_{i}\right), \end{array}$$

where *L*_*i*_ is the loss of cross entropy of the network. Exponentiation of these quantities yields the (un-normalized) likelihoods, and their division performs the normalization so that their probabilities sum to 1. In probabilistic terms, the negative log probability of the accurate class is minimized, which can be regarded as performing maximum likelihood estimation (MLE). A fine feature of this observation is that the regularization term *R* (W) can now be interpreted as the full loss function as approaching from a Gaussian prior over the weight matrix W, where we are executing the maximum a posteriori (MAP) estimation instead of MLE.

## 3. Results and Discussion

### 3.1. Background

In this study, the proposed method is presented using SVM, KNN, NB, and softmax classifiers. The details are shown in Figure 3. The proposed idea is to combine two extracted features, namely, cortical thickness and subcortical volume, from Freesurfer toolbox to differentiate between AD vs other groups. For early prediction, we perform a binary and tertiary classification using the AD, HC, mAD, and aAD datasets to check how well our proposed method has performed on the sMRI 3D image. At the preprocessing stage, normalization was performed for each case. Moreover, the PCA dimensionality reduction technique is utilized to find the optimal number of principal components for each classification, as shown in Figure 4 for AD vs HC. Different numbers of principal components were obtained for different cases. In addition, stratified K-Fold (SKF) cross-validation (CV) was used to confirm the robustness of the classification results. Four different types of classifiers were used to obtain the test sample accuracy, sensitivity, specificity, precision, and F1 score.

### 3.2. Evaluation

To obtain unbiased estimations of the performance, the set of subjects was randomly separated into two parts in a 70/30 ratio for training and testing. The training set was utilized to determine the optimal hyperparameters of each classifier or method and later, to train the classifier. Subsequently, the testing set was used to evaluate the classification performance. The training and testing datasets were identical for all techniques. On the training set, SKF CV was used to estimate the optimal hyperparameters. Two kernel parameters *c* and *γ* for radical basic function SVM and one kernel value *c* for linear SVM were required to be determined according to the Libsvm library. The SVM algorithm performs poorly on the experimental data when the default parameter values are selected. Therefore, the grid search method was utilized to determine the optimal parameters for *c* and *γ* before they were used for training. The pair (*c* and *γ*) with the highest cross-validation accuracy was chosen as the best values. In the present study, the search scales for these two values were set as follows: *c*=1  to  10 and *γ*=(1*e*^−4^, 1*e*^−2^, 0.0001) for all cases.

In addition, the obtained values of *c* and *γ* for all cases are shown in Table 3. For each method, the obtained best-suited optimized value was set to the hyperparameters, which were later utilized to train the classifier using the training groups, and the performance of each resulting classifier was then assessed on the testing set. Thereby, unbiased evaluations of the performances of each method were obtained, whereas for KNN classifier, we have used the elbow method to decide the *k* value. As we know that in the elbow method, if one plots the percentage of variance explained by the cluster against the number of clusters, the first cluster will add much information (explain a lot of variances), but at some point, the marginal gain will drop, giving an angle in the graph. So, the number of clusters is chosen at this point. First, we plot the elbow curve using the Matplotlib library. Moreover, in our case, we provide a range of 1 to 251 for AD vs HC classification problem (which represents the number of subjects for each classification). Therefore, when we plot the graph, the *k* value from the graph where there is a bend tells us how many clusters are there.

The performance of a binary and multiclass classifier can be understood using confusion matrix, as shown in Table 4. The performance of the system was assessed using the SVM, KNN, NB, and softmax classifiers for each precise test including binary and tertiary classification tasks. The diagonal elements of the matrix show the number of correct predictions made by the classifier. These elements can be further separated into true positive (TP) and true negative (TN), thus indicating the correctly identified controls. Similarly, the number of incorrectly classified subjects may be characterized by false negative (FN) and false positive (FP). Accuracy measures the number of examples that were correctly labeled by the classifier, that is,

(7) $$\begin{array}{c} \text{acc}=\frac{\text{TP}+\text{TN}}{\text{TP}+\text{TN}+\text{FP}+\text{FN}}. \end{array}$$

However, for a dataset with unstable class distribution, calculating only the accuracy may result in a misleading estimation of the performance. Therefore, four additional performance metrics should be calculated, namely, specificity, sensitivity, precision, and F1 score. They are defined as follows:

(8) $$\begin{array}{c} \text{sen}=\frac{\text{TP}}{\text{TP}+\text{FN}}, \end{array}$$

(9) $$\begin{array}{c} \text{spe}=\frac{\text{TN}}{\text{TN}+\text{FP}}, \end{array}$$

(10) $$\begin{array}{c} \text{ppv}=\frac{\text{TP}}{\text{TP}+\text{FP}}, \end{array}$$

(11) $$\begin{array}{c} \text{F}1\text{ score}=\frac{2\text{TP}}{2\text{TP}+\text{FP}+\text{FN}}. \end{array}$$

Sensitivity (8) indicates the accuracy of the prediction group, and specificity (9) indicates the accuracy of the prediction of the absence group. Sensitivity measures the success rate for a particular class, i.e., within a class, the percentage of correctly determined subjects (by the classification algorithm) to be in the class. Specificity provides a measure for those not in the class, i.e., it is the percentage of those not in the class that were found not to be in the class. Precision (10) (which is also termed as positive predictive value (PPV)) is the fraction of relevant occurrences among the retrieved occurrences, and F1 score (11) (which is also called F score or F measure) is a quantity related to a test's accuracy.

To evaluate whether each technique performs significantly better than a random classifier, McNemar's chi-squared test was used and its significance level was kept to 0.05, which is a benchmark value. McNemar's test examines the difference between proportions in paired observations. It was used to evaluate the difference between the proportions of accurately classified subjects, i.e., accuracy. The corresponding contingency chart is shown in Table 5.

### 3.3. Classification Results

Both binary and tertiary classification methods were used to measure the classification performance in obtaining cortical and subcortical features.

**Figure 6:**
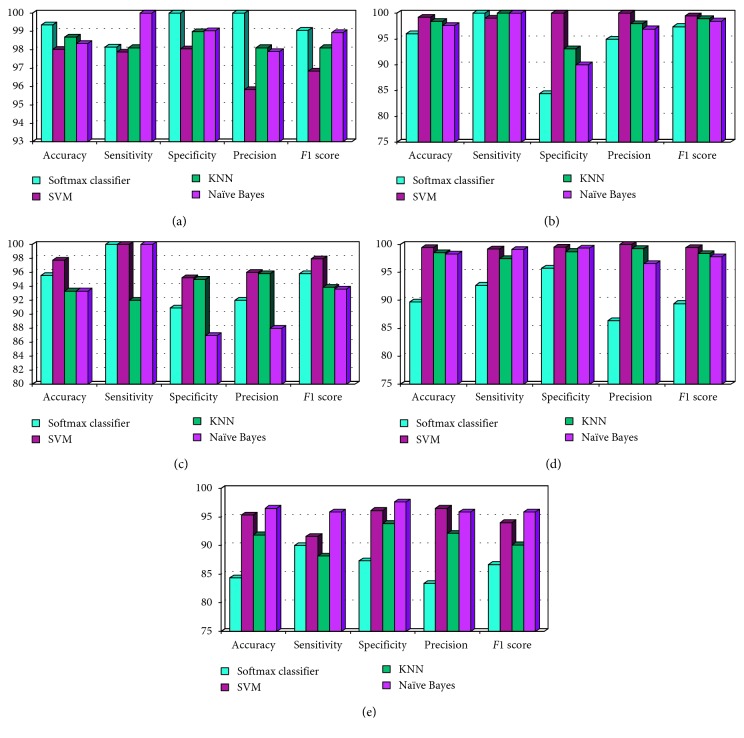
Classification report of each group with the performance measure of accuracy, sensitivity, specificity, precision, and F1 score: (a) AD vs HC, (b) HC vs mAD, (c) mAD vs aAD, (d) AD vs NC vs mAD, and (e) AD vs NC vs aAD.

The obtained results of the classification tests are shown in Tables 6–10 for AD vs HC, HC vs mAD, mAD vs aAD (binary), AD vs HC vs mAD, and AD vs HC vs aAD (tertiary), respectively. The classification report for each case and the performance are shown in Figure 6. All programs were executed in 64-bit Python 3.6 environment on Intel(R) Core(TM) i3-7100 at 3.90 Hz and 8 GB of RAM running Ubuntu 16.04 LTS. The model may be implemented on any computer in which Python 3.6 is compatible.

#### 3.3.1. Binary Classification: AD vs HC, HC vs mAD, and mAD vs aAD

*AD vs HC*. The classification results for AD vs HC are summarized in Table 6 and Figure 6(a). For each case, the dataset was divided into two parts in a 70/30 ratio. All methods performed significantly better, and their *P* value was smaller than the conventional 0.05. The assumption was that there were significant dissimilarities between the two parts. The difference between AD and HC was 64.47% with 95% confidence interval from 56.87% to 72.08%, and it was highly significant (*P* < 0.000001). Softmax classified AD vs HC with a high accuracy of 99.34, sensitivity of 98.14, specificity of 100, precision of 100, and F1 score of 99.06.

*HC vs mAD.* The classification report for HC vs mAD is summarized in Table 7 and Figure 6(b). In this case, all techniques achieved significantly better *P* value than chance (*P* < 0.05); however, SVM classified HC vs mAD highly significantly (*P* < 0.000001) with an accuracy of 99.2, sensitivity of 99.02, specificity of 100, precision of 100, and F1 score of 99.51.

*mAD vs aAD.* The classification results for mAD vs aAD are summarized in Table 8 and Figure 6(c). Here, three methods performed significantly better than chance, and their *p* value was less than the conventional value (*P* < 0.05); however, KNN had a *P* value greater than the conventional value. SVM had a *P* value significantly better than the other three methods and classified mAD vs aAD with a high accuracy of 97.77, sensitivity of 100, specificity of 95.23, precision of 96, and F1 score of 97.95.

#### 3.3.2. Multiclass Classification

For multiclass classification, accuracy may be a misleading indication of the performance; thus, in this case, the F1 score metric is particularly useful.

*AD vs HC vs mAD.* The classification results for AD vs HC vs mAD are summarized in Table 9, and the plot is shown in Figure 6(d). In this case, SVM classified AD vs HC vs mAD with an accuracy of 99.42, sensitivity of 99.18, specificity of 99.5, precision of 99.99, and F1 score of 99.43. The F1 score indicates a high correct percentage; it can be claimed that SVM classified AD vs HC vs mAD more accurately.

*AD vs HC vs aAD.* The classification results for AD vs HC vs aAD are summarized in Table 10 and are shown in Figure 6(d). It can be seen that NB classified the multiclass case significantly more accurately, with an accuracy of 96.53, sensitivity of 95.88, specificity of 97.64, precision of 95.88, and F1 score of 95.88.

#### 3.3.3. Comparing with Others' State-of-the-Art Methods

As we know the NRCD dataset is not available publicly, that is why we apply our proposed method on the OASIS dataset which can be downloaded from (https://www.oasis-brains.org/). The OASIS database was launched in 2007 as a public-private partnership. OASIS provides brain-imaging dataset, which is freely available for sharing and data study. This dataset consists of a cross-sectional group of 416 patients, which covers the adult lifespan aged from 18 to 96 including individuals with early-phase Alzheimer's disease (AD). The subjects are right-handed, and they include both men and women. From 416 subjects, 100 included subjects above the age of 60 have been identified with the very mild-to-mild AD and the remaining 316 are diagnosed as normal. All structural sMRI scans used in this experiment were acquired from 1.5T scanners.

At first, we extracted 110 (cortical and subcortical) features from all 416 subjects using the same Freesurfer version which is used for the NRCD dataset. After that, we followed the same procedure that we had applied for the NRCD dataset. As can be seen from Table 11, all methods performed significantly better, and their *P* value was smaller than the conventional 0.05. The assumption was that there were significant dissimilarities between these two parts. Our proposed method achieved a better result with 98.40% of accuracy, 93.75% of sensitivity, 100% of specificity, 100% of precision, and 96.77% of F1 score with softmax classifier. It can also be seen that SVM follows the softmax classifier in terms of performance.

**Figure 7:**
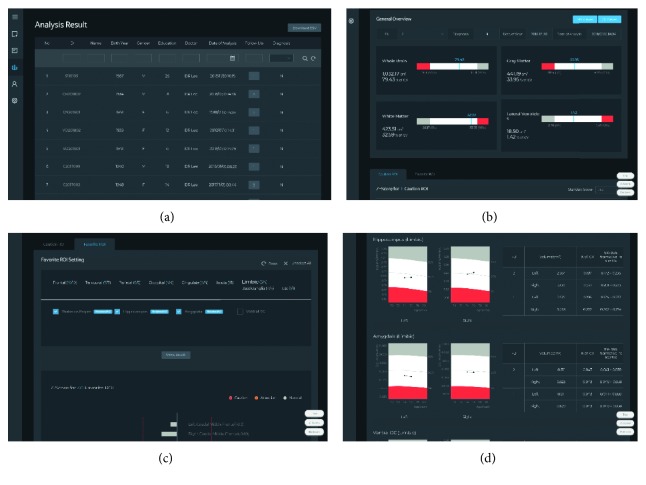
Comparison between the proposed results and conventional image analysis tool [27]: (a) list of subjects, (b) measuring the whole brain, gray matter, white matter, and lateral ventricle by the standard brain map, (c) setting the ROI, and (d) volumetric and ICV regarding ROIs.

To further demonstrate the usefulness of our proposed method, we compared it with 9 state-of-the-art approaches as shown in Table 12. The result in Table 12 shows that Jack et al. [11] used the kernel-LICA-DC method to classify the AD vs HC and achieved a classification accuracy of 74.25%, a sensitivity of 96%, and a specificity of 52.5%. Jha et al. [19] used (deep learning method) sparse autoencoder technique to classify the classification problem and achieved a classification accuracy of 91.6%, a sensitivity of 98.09%, and a specificity of 84.09%. In both of these cases, they have achieved decent accuracy but their specificity is very low compared to our proposed method. Likewise, Islam and Zhang [20] used an ensemble of deep convolutional neural networks technique and achieved a classification accuracy of 93.18% and a specificity of 93%. Farhan et al. [21] used an ensemble of a classifier for the classification of AD vs HC and achieved a classification accuracy of 93.75%, a sensitivity of 100%, and a specificity of 87.5%. Despite using deep learning and ensemble classifier methods, their accuracy, sensitivity, and specificity are low compared to our proposed method. Khajehnejad et al. [18] used the semisupervised technique for classification and achieved an accuracy of 93.86%, a sensitivity of 94.65%, and a specificity of 93.22%. Jha et al. [16] used the extreme learning machine method for classification of AD vs HC with dual-tree wavelet principal coefficients and achieved an accuracy of 95.27%, a sensitivity of 96.59% and a specificity of 93.03%. And, Lama et al. [22] used cortical features for early detection of AD and RELM for classification and achieved an accuracy of 77.88%, a sensitivity of 68.85%, and a specificity of 83.54%. Despite using new methods for classification, their classification performance is very low compared to our results as shown in Table 12. As can be seen from Table 12, our proposed method using softmax classifier has outperformed all state-of-the-art methods and achieved an accuracy of 99.34%, a sensitivity of 98.14%, and a specificity of 100% for the NRCD dataset, and for the OASIS dataset, our proposed method achieved an accuracy of 98.40%, a sensitivity of 93.75%, and a specificity of 100%. Additionally, to enhance the performance results, we have compared with the performance results of the proposed method and the image analysis system [27] as shown in Figure 7.

## 4. Discussion

In this study, different techniques for the classification of subjects with AD and mAD based on anatomical T1-weighted MRI were compared. To assess and compare their performance, five experiments were conducted: AD vs HC, HC vs mAD, mAD vs aAD (binary classification), AD vs HC vs mAD, and AD vs HC vs aAD (multiclass classification). The set of subjects was randomly split into two groups in the ratio of 70/30 for training and testing. Then, PCA was applied for dimensionality reduction. Subsequently, four classifiers were used to evaluate performance. For the SVM classifier, the optimal parameter value was determined by using a grid search and SKF CV. Then, those values were used to train the SVM classifier using the training dataset, and the testing dataset was evaluated on the training model to evaluate the performance. Overall, the radical basic function (RBF) SVM classifier yielded better result compared with the other methods in several cases. Regarding the softmax classifier, Adam optimization was used with learning rate 1*e*^−2^, 1000 epochs, and batch size 50, and it yielded highly satisfactory results for binary classification, particularly for AD vs HC with an accuracy of 99.34.

## 5. Conclusions

A new method for automatic classification of an AD from MCI (asymptomatic or converted to the AD) and a healthy group is proposed using combined features extracted from an automated toolbox, where sMRI scans of NRCD dataset is used as an input image. Here, four different types of classifiers (softmax classifier, SVM, KNN, and naïve Bayes) were used to classify five different types of the classification problem. These experimental results confirm that the proposed method had effectively predicted future clinical changes between (mAD vs aAD) patients, as can be seen in Table 8. Our proposed model has achieved 97.99% of accuracy, 100% of sensitivity, 95.23% of specificity, 96% of precision, and F1 score as 97.95% using the RBF-SVM classifier for mAD vs aAD classification. Efficient and reliable outcomes were obtained, which prove the effectiveness of the proposed technique. Comparison of the results showed that the RBF-SVM classifier achieved highly satisfactory performance for three cases (HC vs mAD, mAD vs aAD, and AD vs HC vs mAD), where the NB classifier was suitable for predicting AD vs HC vs aAD. As we can say that, our proposed technique to combine cortical thickness and subcortical volume features of the same subjects for classification of a binary and tertiary group performed very well in our dataset as well as in the OASIS dataset.

In the present study, only cortical thickness and subcortical volume features were considered for the classification process. However, in the future, other different types of features will be used, such as the hippocampus and amygdala for the classification of the AD. Moreover, longitudinal datasets will be used for studying the changes over time between AD, mAD, and aAD. In this study, only NRCD dataset was used for research, so in future, we are going to compare our model with differently available datasets for early prediction of the AD.

## Figures and Tables

**Table 1:** Demographic characteristics of the studied population (from the NRCD database).

Group	Number of subjects	Age	Gender		Education
			M	F	
AD	81	71.86 ± 7.09 [56–83]	39	42	7.34 ± 4.88 [0–18]
mAD	39	73.23 ± 7.34 [49–87]	25	14	8.20 ± 5.19 [0–18]
aAD	35	72.74 ± 4.82 [61–83]	15	20	7.88 ± 6.30 [0–18]
HC	171	71.66 ± 5.43 [60–85]	83	88	9.16 ± 5.54 [0–22]

Values are specified as mean ± standard deviation [range].

**Table 2:** Features used in this study.

Methods	Number of features	Descriptions
Cortical thickness and subcortical volume segmentation	110	Based on group-level statistical analysis measure of subcortical segmentation volume and cortical thickness

**Table 3:** Best obtained *c* and *γ* values using the grid search method.

Group	*C* value	Gamma value
AD vs HC	1	0.0025
HC vs mAD	1	0.005600000000000001
mAD vs aAD	1	0.005
AD vs HC vs mAD	6	0.0096
AD vs HC vs aAD	3	0.0088

**Table 4:** Confusion matrix.

True class	Predicted class	
	G2	G2
G1	TP	FN
G1	FP	TN

**Table 5:** Contingency table for the McNemar's test.

	Group 2: correctly classified	Group 2: misclassified
Group 1: correctly classified	A	B
Group 1: misclassified	C	D

**Table 6:** Classification results for AD vs HC.

AD vs HC	ACC	SEN	SPEC	PRE	F1 score	McNemar's test
Softmax classifier	**99.34**	98.14	**100**	**100**	**99.06**	*P* < 0.000001
SVM	98.02	97.87	98.05	95.83	96.84	*P* < 0.000001
KNN	98.68	98.11	98.98	98.11	98.11	*P* < 0.000001
Naïve Bayes	98.40	**100**	99.04	97.91	98.94	*P* < 0.000001

**Table 7:** Classification results for HC vs mAD.

HC vs mAD	ACC	SEN	SPEC	PRE	F1 score	McNemar's test
Softmax classifier	96.03	**100**	84.37	94.94	97.4	*P*=0.000113
SVM	**99.2**	99.02	**100**	**100**	**99.51**	*P* < 0.000001
KNN	98.41	**100**	93.1	97.97	98.97	*P*=0.000002
Naïve Bayes	97.61	**100**	90	96.96	98.46	*P*=0.000008

**Table 8:** Classification results for mAD and aAD.

mAD vs aAD	ACC	SEN	SPEC	PRE	F1 score	McNemar's test
Softmax classifier	95.55	100	90.9	92	95.83	*P*=0.000121
SVM	**97.77**	**100**	**95.23**	**96**	**97.95**	*P* < 0.000001
KNN	93.33	92	95	95.83	93.87	*P*=1
Naïve Bayes	93.33	100	86.95	88	93.61	*P*=0.000488

**Table 9:** Classification results for AD vs HC vs mAD.

AD vs HC vs mAD	ACC	SEN	SPEC	PRE	F1 score
Softmax classifier	89.71	92.67	95.76	86.38	89.42
SVM	**99.42**	**99.18**	**99.5**	**99.99**	**99.43**
KNN	98.52	97.46	98.7	99.23	98.33
Naïve Bayes	98.28	99.07	99.32	96.55	97.79

**Table 10:** Classification results of AD vs HC vs aAD.

AD vs HC vs mAD	ACC	SEN	SPEC	PRE	F1 score
Softmax classifier	84.39	90	87.35	83.36	86.69
SVM	95.37	91.63	96.16	**96.56**	94.03
KNN	91.9	88.17	93.86	92.15	90.12
Naïve Bayes	**96.53**	**95.88**	**97.64**	95.88	**95.88**

**Table 11:** Classification results for AD vs HC.

AD vs HC	ACC	SEN	SPEC	PRE	F1 score	McNemar's test
Softmax classifier	**98.40**	93.75	**100**	**100**	**96.77**	*P* < 0.000001
SVM	97.60	**96.55**	97.91	93.33	94.91	*P* < 0.000001
KNN	88.70	96.0	85.17	87.80	82.70	*P* < 0.000001
Naïve Bayes	94.40	86.36	96.11	82.60	84.44	*P* < 0.000001

**Table 12:** Algorithm performance comparison over OASIS and ADNI MRI data.

Approach	Year	Dataset	Classifier	Modalities	AD vs HC		
					ACC	SEN	SPEC
Cuingnet et al. [23]	2011	ADNI	SVM	MRI	NA	81%	95%
Cho et al. [25]	2012	ADNI	LDA	MRI	NA	82%	93%
Chyzhyk et al. [17]	2012	OASIS	Kernel-LICA-DC	MRI	74.25	96	52.5
Lama et al. [22]	2017	ADNI	RELM	MRI	77.88	68.85	83.54
Jha and Kwon [19]	2017	OASIS	Sparse autoencoder	MRI	91.6	98.09	84.09
Islam and Zhang [20]	2017	OASIS	Ensemble of deep convolutional neural networks	MRI	93.18	NA	93
Farhan et al. [21]	2014	OASIS	Ensemble of classifier	MRI	93.75	**100**	87.5
Khajehnejad et al. [18]	2017	OASIS	Semisupervised classifier	MRI	93.86	94.65	93.22
Jha et al. [16]	2018	ADNI	ELM	MRI	90.26	90.27	90.20
		OASIS			95.27	96.59	93.03
Proposed method	2018	NRCD	Softmax classifier	MRI	**99.34**	**98.14**	**100**
	2018	OASIS	Softmax classifier	MRI	**98.40**	93.75	**100**

## Data Availability

The National Research Center for Dementia (NRCD) dataset was used to support the findings of this study. At first, we would like to say that NRCD is a private dataset which was generated in Chosun University hospitals, and it originally belongs to Chosun University. We cannot share it or open it online for others due to privacy reasons. Later, to compare with other recent state-of-the-art methods, we have used OASIS dataset which was downloaded from https://www.oasisbrains.org/.
